# Heterozygous variants in the teashirt zinc finger homeobox 3 (*TSHZ3*) gene in human congenital anomalies of the kidney and urinary tract

**DOI:** 10.1038/s41431-024-01710-y

**Published:** 2024-10-17

**Authors:** Esra Kesdiren, Helge Martens, Frank Brand, Lina Werfel, Lukas Wedekind, Mark-Oliver Trowe, Jessica Schmitz, Imke Hennies, Robert Geffers, Zoran Gucev, Tomáš Seeman, Sonja Schmidt, Velibor Tasic, Laurent Fasano, Jan H. Bräsen, Andreas Kispert, Anne Christians, Dieter Haffner, Ruthild G. Weber

**Affiliations:** 1 Department of Human Genetics, Hannover Medical School, Hannover, Germany; 2 Department of Pediatric Kidney, Liver, Metabolic and Neurological Diseases, Hannover Medical School, Hannover, Germany; 3 Institute of Molecular Biology, Hannover Medical School, Hannover, Germany; 4 Nephropathology, Department of Pathology, Hannover Medical School, Hannover, Germany; 5 Genome Analytics Research Group, Helmholtz Centre for Infection Research, Braunschweig, Germany; 6 Pediatric Nephrology, University Children’s Hospital, Skopje, Macedonia; 7 Department of Pediatrics, 2nd Faculty of Medicine, Charles University, Prague, Czech Republic; 8 Department of Pediatrics, Faculty of Medicine, University of Ostrava, Ostrava, Czech Republic; 9 Department of General, Visceral and Pediatric Surgery, University Medical Center Göttingen, Göttingen, Germany; 10 Aix-Marseille Univ, CNRS, IBDM UMR7288, Marseille, France

**Keywords:** Genetics research, Risk factors, Paediatric kidney disease

## Abstract

Around 180 genes have been associated with congenital anomalies of the kidney and urinary tract (CAKUT) in mice, and represent promising novel candidate genes for human CAKUT. In whole-exome sequencing data of two siblings with genetically unresolved multicystic dysplastic kidneys (MCDK), prioritizing variants in murine CAKUT-associated genes yielded a rare variant in the teashirt zinc finger homeobox 3 (*TSHZ3*) gene. Therefore, the role of *TSHZ3* in human CAKUT was assessed. Twelve CAKUT patients from 9/301 (3%) families carried five different rare heterozygous *TSHZ3* missense variants predicted to be deleterious. CAKUT patients with versus without *TSHZ3* variants were more likely to present with hydronephrosis, hydroureter, ureteropelvic junction obstruction, MCDK, and with genital anomalies, developmental delay, overlapping with the previously described phenotypes in *Tshz3*-mutant mice and patients with heterozygous 19q12-q13.11 deletions encompassing the *TSHZ3* locus. Comparable with *Tshz3*-mutant mice, the smooth muscle layer was disorganized in the renal pelvis and thinner in the proximal ureter of the nephrectomy specimen of a *TSHZ3* variant carrier compared to controls. *TSHZ3* was expressed in the human fetal kidney, and strongly at embryonic day 11.5-14.5 in mesenchymal compartments of the murine ureter, kidney, and bladder. *TSHZ3* variants in a 5′ region were more frequent in CAKUT patients than in gnomAD samples (*p* < 0.001). Mutant TSHZ3 harboring N-terminal variants showed significantly altered SOX9 and/or myocardin binding, possibly adversely affecting smooth muscle differentiation. Our results provide evidence that heterozygous *TSHZ3* variants are associated with human CAKUT, particularly MCDK, hydronephrosis, and hydroureter, and, inconsistently, with specific extrarenal features, including genital anomalies.

## Introduction

Congenital anomalies of the kidney and urinary tract (CAKUT) arise as a result of spatial and temporal dysregulation during the morphogenesis of the kidney and urinary tract. Kidney anomalies include a missing kidney (agenesis), a kidney with multiple cysts and no function (multicystic dysplastic kidney, MCDK), a kidney with abnormal shape and differentiation (dysplasia), and a small kidney with reduced number of nephrons (hypoplasia). These kidney malformations as well as a dilated kidney pelvis (hydronephrosis) and ureter (hydroureter) can be isolated anomalies or occur in conjunction with other CAKUT phenotypes. With a prevalence of 3–9/1000 live births, CAKUT account for approximately 40% of pediatric cases with end-stage kidney disease [1–3]. Extrarenal anomalies are observed in around one-third of CAKUT patients [4]. A family history of CAKUT is reported in around 15% of patients, with autosomal dominant inheritance predominating in familial cases.

Mouse models help to elucidate the molecular mechanisms underlying CAKUT [5, 6]. Moreover, the around 180 monogenic mouse models that exhibit CAKUT may provide leads for identifying novel human CAKUT-associated genes [5, 7]. To date, around 60 genes are known to cause monogenic CAKUT if mutated [8–11]. Striving to identify new CAKUT-associated genes is motivated by the fact that a genetic etiology is currently detected, on average, in only 16% of CAKUT cases [10], although the diagnostic yield is higher in specific CAKUT cohorts, such as CAKUT patients diagnosed in the first thousand days of live with kidney and extrarenal anomalies (41%) or requiring kidney replacement therapy before 3 years of age (43%) [12]. Gene discovery has been facilitated by applying next-generation sequencing techniques to the study of large CAKUT families with seven or more affected individuals leading to the identification of novel CAKUT-associated genes, such as *DSTYK* [13], *TBX18* [14], and *GREB1L* [15]. However, investigating CAKUT families with one or two affected individuals including the de novo analysis of parent-patient-trios in sporadic CAKUT patients has also been successful in detecting CAKUT-related genes, such as *PBX1* [16], *ROBO1* [17], *TBC1D1 *[12, 18], and *LIFR* [12,19].

While variable expressivity is common in CAKUT, we selected a rare family with the same CAKUT phenotype, i.e., MCDK, in two siblings, and applied whole-exome sequencing (WES) and a linkage- and candidate-based analysis strategy. In both siblings, we identified a rare missense variant in the teashirt zinc finger homeobox 3 (*TSHZ3*) gene, a known murine CAKUT-associated gene [20, 21], variants of which were previously reported in two CAKUT patients [22, 23]. *TSHZ3* encodes a transcription factor regulating smooth muscle (SM) cell differentiation via interaction with SRY-box transcription factor 9 (SOX9) and myocardin (MYOCD) during ureter (and kidney) development [20, 24–26]. By analyzing the frequency of *TSHZ3* variants and their associated phenotype spectrum in a cohort of 301 CAKUT families, determining *TSHZ3* expression in human fetal and adult tissues as well as the *Tshz3* expression pattern during murine development, and assessing the functional consequences of mutant TSHZ3 harboring N-terminal variants, we provide evidence that rare *TSHZ3* missense variants may impact SM cell differentiation via altered SOX9 and MYOCD binding, leading to CAKUT, in particular MCDK, hydronephrosis and hydroureter, as well as specific extrarenal features in humans.

## Materials and methods

### Patients

The study was approved by the Ethics Committees of Hannover Medical School, Hannover, Germany, and Skopje University Hospital, Skopje, North Macedonia. Each family provided informed consent for participation in the study. Of the 313 analyzed CAKUT patients from 301 families, 202 were male, 111 were female, and their mean age was 13.8 years (range 3–52 years). The spectrum of kidney and/or urinary tract anomalies of the analyzed patients is listed in Supplementary Table 1. Patients who only had vesicoureteral reflux (VUR) were excluded. Case reports of CAKUT patients carrying *TSHZ3* variants are provided in the results section and the supplementary material.

### Whole-exome and targeted *TSHZ3* sequencing

WES was performed on leukocyte DNA of 55 CAKUT index patients, 18 family members, and 153 adults without clinical signs of impaired kidney health serving as controls using the SureSelectXT Human All Exon V4, V5 or V5+UTRs target enrichment kit (all Agilent, Santa Clara, CA, USA) on a HiSeq 2000 or 2500 or a NovaSeq 6000 (all Illumina, San Diego, CA, USA) sequencer. All samples were sequenced to a mean coverage of 50x. Sequencing data were aligned to the human reference genome (GRCh37/hg19), variations were called using QIAGEN CLC Genomic Workbench (Qiagen, Hilden, Germany), annotated and prioritized using QIAGEN Clinical Insight Interpret Translational (Qiagen), and our in-house data analysis workflow. Supplementary Table 2 summarizes the linkage- and candidate-based strategy used to analyze WES data from two affected siblings of index family F004. Using conventional chain termination protocols, (i) mutational analysis of all coding exons and adjacent intronic regions of the *TSHZ3* gene (NM_020856.4) was done in 246 additional CAKUT index patients and 11 family members, (ii) selected variants identified by WES analysis were verified, and (iii) familial segregation was determined on a 3130XL Genetic Analyzer (Thermo Fisher Scientific, Waltham, MA, USA; oligonucleotide sequences are listed in Supplementary Table 3). Minor allele frequencies (MAF) were retrieved from the Genome Aggregation Database (gnomAD) [27]. Variant pathogenicity was predicted using CADD, MutationTaster, SIFT, PROVEAN, PolyPhen-2, and classified according to the American College of Medical Genetics and Genomics and the Association for Molecular Pathology (ACMG/AMP) guidelines [28]. Multiple sequence alignment was created using the ClustalW sequence alignment program [29]. The phylogenetic tree was generated using iTol [30].

### Animals

All experiments were approved by the Ethics Committee of the Lower Saxony State Office for Consumer Protection and Food Safety. Murine embryos were derived from matings of ZtmHan:NMRI wild-type mice. For timed pregnancies, vaginal plugs were checked in the morning after mating, and noon was considered as embryonic day (E) 0.5. Embryos and urogenital systems were dissected in phosphate-buffered saline (PBS), fixed in 4% paraformaldehyde in PBS, dehydrated in methanol, and stored in 100% methanol at −20 °C prior to RNA in situ hybridization.

### Immunohistochemistry, quantitative analysis of *TSHZ3* mRNA expression, RNA in situ hybridization, cloning of expression constructs and site-directed mutagenesis, cell culture and transient transfection, immunoprecipitation, Western blot analysis

Procedures are described in the supplementary material.

### Statistical analysis

Statistical analysis was performed using MATLAB and Statistics Toolbox Release 2022b (The MathWorks, Natick, MA, USA). Student’s *t*-test or Fisher’s exact test (two-tailed) were used, as appropriate, whereby *p* values of <0.05 were considered significant.

## Results

### Rare heterozygous *TSHZ3* missense variants predicted to be deleterious were identified in 12 CAKUT patients from nine of 301 (3%) families

**Fig. 1:**
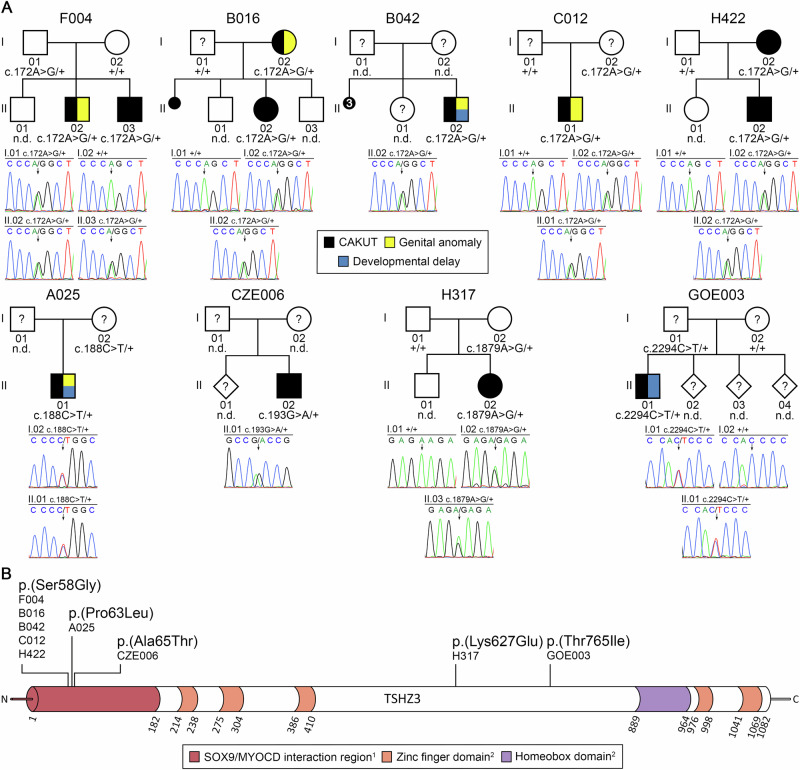
Whole-exome and targeted sequencing yielded five different rare (MAF < 0.002) heterozygous *TSHZ3* missense variants predicted to be deleterious in 12 CAKUT patients from 9 of 301 (3%) families. **A** Pedigrees of the nine families and electropherograms of the *TSHZ3* variants (affected base positions are indicated by arrows) in all tested family members are shown. In pedigrees, squares denote males, circles females, rhombs unknown gender, small black circles spontaneous abortions, and colored symbols affected individuals with phenotypes as indicated. Six of 12 (50%) CAKUT patients carrying *TSHZ3* variants presented with genital anomalies and/or developmental delay upon reverse phenotyping. Question marks denote family members with no clinical information available or without kidney ultrasound. **B** Scheme showing the localization of affected amino acid residues within the TSHZ3 protein. Three of the five variants are located within an N-terminal region (amino acids 1-182 of TSHZ3) reported to mediate SOX9/MYOCD interaction [24]. *+* *TSHZ3* wild-type sequence, *n.d.* individual with non-available DNA. ^1^according to Martin et al. [24]; ^2^according to InterPro (http://www.ebi.ac.uk/interpro/protein/UniProt/Q63HK5/).

In the index family F004, two male siblings (F004-II.02 and F004-II.03) of non-consanguineous German parents were prenatally diagnosed with the same CAKUT phenotype, i.e., unilateral MCDK (Fig. 1A, Table 1). Patient F004-II.02 was born prematurely at 33 weeks of gestation, and was additionally diagnosed with bilateral cryptorchidism and phimosis. In both siblings, postnatal ultrasound revealed compensatory hypertrophy of the contralateral kidney. Kidney ultrasound of the parents and an older brother were unremarkable. WES performed on the leukocyte DNA of both affected siblings was analyzed using a linkage- and candidate-based strategy. By prioritizing high-quality, non-silent, rare (MAF ≤ 0.002) variants not present in in-house controls, and predicted to be deleterious, 23 variants shared by both siblings were identified. While none of the 23 variants was located in a gene from our in-house list of 279 (candidate) genes associated with CAKUT in humans, one variant, NM_020856.4(*TSHZ3*):c.172A>G p.(Ser58Gly), was located in a murine CAKUT-associated gene (Supplementary Table 2). The *TSHZ3* variant was confirmed by Sanger sequencing to be heterozygous and was inherited from the patients’ father (Fig. 1A).

**Table 1:** Rare (MAF < 0.002) heterozygous *TSHZ3* variants and phenotypes detected in 12 CAKUT patients from 9 of 301 (3%) families.

Chr. position^a^/dbSNP ID	Nucleotide alteration, deduced protein change^a^	MAF^b^	Variant frequency comparison in our families vs. controls^c^	Pathogenicity predictions^d^: CADD/ Mutation Taster/ SIFT/ PROVEAN/ PolyPhen-2	Clinical interpretation according to ACMG/AMP guidelines^e^	Family, country of origin	Case, gender, year of birth	Inheritance	CAKUT phenotypes^f^	Congenital extrarenal anomalies^f^		
										Neurological anomalies	Genital anomalies	Other
19:31279621/rs201390565	c.172A>G p.(Ser58Gly)	0.0008734	*p* = 0.0002	21.6/DC/T/N/B	Likely pathogenic (PS4, PS3_ moderate, PM1, BP4)	F004, Germany	II.02, male, 2010	Pat	MCDK (r)	−	Cryptorchidism (r + l), phimosis	Preterm birth (33 + 4 weeks of gestation)
							II.03, male, 2015^g^		MCDK (l)	−	−	−
						B016, Turkey	II.02, female, 2007	Mat	Kidney hypodysplasia (r + l)	−	−	Preterm birth (29 + 0 weeks of gestation), persistent ductus arteriosus, atrial septal defect, hemangioma
							I.02, female, 1970	n.d.	Kidney cysts (r + l)	−	Ovarian cyst	−
						B042, Germany	II.02, male, 2011^g^	n.d.	MCDK (r), hydronephrosis and hydroureter (r + l), duplex ureter (r), VUR grade III (l), PUV	Hypoxic ischemic encephalopathy, developmental delay/intellectual disability, epilepsy	Cryptorchidism (r + l)	Preterm birth (36 + 3 weeks of gestation), microcephaly, muscle hypotonia, pulmonary hypoplasia, neurogenic bladder dysfunction, pes calcaneus (r + l)
						C012, Germany	II.01, male, 2015^g^	Mat	Hydronephrosis and hydroureter (l), VUR grade V (r + l)	−	Phimosis	−
						H422, North Macedonia	II.02, male, 2016	Mat	Hydronephrosis and ureteropelvic junction obstruction (l)	−	−	Clinodactyly (toe IV, V) (r + l)
							I.02, female, 1978	n.d.	Non-obstructive duplex kidney (r)	−	−	−
19:31279605/rs779284228	c.188C>T p.(Pro63Leu)	0.00004778	*p* = 0.0287	25.1/DC/D/Del/proD	Likely pathogenic (PS4, PS3_ moderate, PM1, PP3)	A025, Germany	II.01, male, 2008	Mat	Kidney hypodysplasia, hydronephrosis, hydroureter, VUR grade IV (r + l)	Developmental delay	Retractile testis (l)	Postnatal growth retardation, growth hormone deficiency
19:31279600/rs111618794	c.193G>A p.(Ala65Thr)	0.001299	*p* = 0.5432	22.9/DC/D/N/proD	Uncertain significance (PM1, PP3)	CZE006, Czech Republic	II.02, male, 2008^g^	n.d.	Hydronephrosis, hydroureter, VUR grade IV–V (r + l), PUV	−	−	−
19:31277914/rs768599058	c.1879A>G p.(Lys627Glu)	0.00001611	*p* = 0.01	24.4/DC/T/N/proD	Uncertain significance (PS4, PP3)	H317, North Macedonia	II.02, female, 2013	Mat	Horseshoe kidney	−	−	High-arched palate, ear helix anomaly (r), single transverse palmar crease (l)
19:31277499/rs770277792	c.2294C>T p.(Thr765Ile)	0.00001865	*p* = 0.0115	23.3/DC/D/N/posD	Uncertain significance (PS4, PP3)	GOE003, Germany	II.01, male, 2003^g^	Pat	Duplex kidney, kidney dysplasia of the lower pole, hydronephrosis, ureteropelvic junction obstruction (l)	Developmental delay/intellectual disability, dilated lateral ventricles, mega cisterna magna, pineal gland cyst	−	Pes planus (r + l)

*B* benign, *D* damaging, *DC* disease-causing, *Del* deleterious, *l* left, *MAF* minor allele frequency, *Mat* maternal, *MCDK* multicystic dysplastic kidney, *N* neutral, *n.d.* not determined, *Pat* paternal, *posD* possibly damaging, *proD* probably damaging, *PUV* posterior urethral valves, *r* right, *T* tolerated, *VUR* vesicoureteral reflux.

^a^Reference sequence used: NM_020856.4, NP_065907.2, genome build GRCh38/hg38. Variants were submitted to ClinVar (accession numbers SCV004803225 - SCV004803229, http://www.ncbi.nlm.nih.gov/clinvar/).

^b^MAF according to gnomAD v4.1.0, total population (https://gnomad.broadinstitute.org/).

^c^The variant frequency in our cohort (*n* = 301 families) was compared to gnomAD v4.1.0, total population; *p* values were calculated using the two-tailed Fisher’s exact test.

^d^Pathogenicity prediction according to CADD: ≥20 considered pathogenic (https://cadd.gs.washington.edu/snv)/MutationTaster (http://www.mutationtaster.org/)/SIFT (https://sift.bii.a-star.edu.sg)/PROVEAN (https://www.jcvi.org/research/provean)/PolyPhen-2 (http://genetics.bwh.harvard.edu/pph2/).

^e^According to Richards et al. [28]; also considering biochemical variant characterization (Fig. 5), whereby PS3 was rated as moderate.

**Fig. 2:**
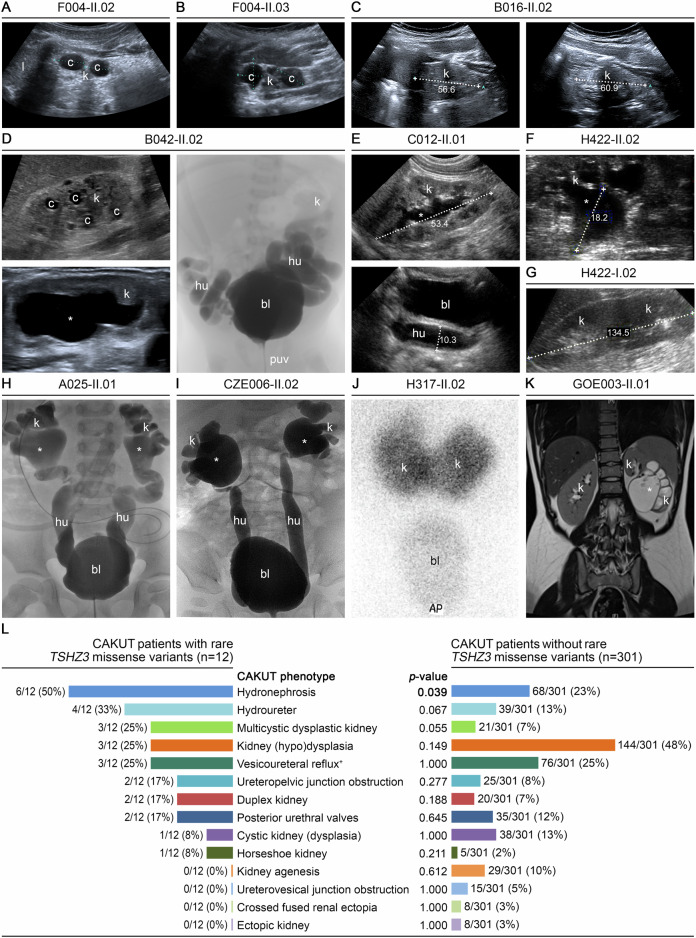
CAKUT phenotypes of patients carrying *TSHZ3* variants, and comparison of CAKUT phenotype frequency in patients with and without *TSHZ3* variants. **A** Patient II.02 from index family F004 was diagnosed with right-sided MCDK by ultrasound (US). **B** Kidney US of his brother, F004-II.03, who presented with left-sided MCDK. **C** Patient B016-II.02 showing hypodysplasia of both kidneys by US. **D** Kidney US revealed right-sided MCDK and left-sided hydronephrosis in patient B042-II.02. Voiding cystourethrography (VCUG) showing bilateral hydroureters and posterior urethral valves. **E** Kidney US of patient C012-II.01 showing left-sided hydronephrosis and hydroureter. **F** Patient H422-II.02 was diagnosed with left-sided hydronephrosis, as shown by US, and ureteropelvic junction obstruction. **G** Kidney US of his mother, H422-I.02, showing right-sided non-obstructive duplex kidney. **H** By kidney US (not shown) and VCUG, patient A025-II.01 was diagnosed with bilateral kidney hypodysplasia, hydronephrosis, hydroureters, and VUR grade IV. **I** VCUG of patient CZE006-II.02 revealing bilateral hydronephrosis, hydroureters, VUR grade IV–V, and posterior urethral valves. **J** A dimercaptosuccinic acid kidney scan in patient H317-II.02 showing a horseshoe kidney. **K** Magnetic resonance imaging of patient GOE003-II.01 revealing left-sided duplex kidney, kidney dysplasia of the lower pole, hydronephrosis, and ureteropelvic junction obstruction of the lower kidney. **L** Frequency of CAKUT phenotypes (colored bars) in patients with rare heterozygous *TSHZ3* variants (*n* = 12) compared to CAKUT patients without *TSHZ3* variants (*n* = 301). Hydronephrosis (*p* = 0.039), hydroureter (*p* = 0.067), and MCDK (*p* = 0.055) were (significantly) more frequent in CAKUT patients with versus without *TSHZ3* variants. ^*+*^ VUR was never isolated, but occurred in conjunction with structural kidney or urinary tract defects in patients analyzed here, * hydronephrosis, AP anterior posterior, bl bladder, c cyst, hu hydroureter, k kidney, l liver, puv posterior urethral valves, VUR vesicoureteral reflux.

^f^Selected phenotypes are shown in Fig. 2, case reports are provided in the results section and supplementary material.

^g^Likely pathogenic or pathogenic variants in other human CAKUT-associated genes were ruled out by WES.

To determine the frequency of *TSHZ3* variants in a larger cohort of CAKUT patients, 300 additional CAKUT families (phenotype spectrum listed in Supplementary Table 1) were analyzed by WES or targeted *TSHZ3* sequencing. We identified five different rare (MAF < 0.002) missense variants in eight other CAKUT families (Fig. 1A, Table 1, case reports are provided in the supplementary material). The c.172A>G p.(Ser58Gly) variant identified in F004 was detected in four other families, and co-segregation with CAKUT could be shown in a total of three families, i.e., F004, B016, and H422 (some members of families B042 and C012 were not available for genetic testing and/or phenotypic evaluation). The four other variants were identified in one family each. *TSHZ3* variants were inherited maternally in 5/9 families, paternally in 2/9 families, and inheritance could not be determined in 2/9 families (Fig. 1A, Table 1). All variants have a CADD score >20, indicating that they are in the top 1% of most deleterious variants in the human genome (Table 1), and all are located in a region of high genomic constraint according to gnomAD v4.1.0, with a regional genomic constraint of 6.36 (values range from −10 to 10) for the c.172A>G p.(Ser58Gly), c.188C>T p.(Pro63Leu), and c.193G>A p.(Ala65Thr) variants, and of 4.51 for the c.1879A>G p.(Lys627Glu) and c.2294C>T p.(Thr765Ile) variants. Three variants, i.e., c.172A>G p.(Ser58Gly), c.188C>T p.(Pro63Leu), and c.193G>A p.(Ala65Thr), encode amino acids located in an N-terminal region of TSHZ3, i.e., amino acids 1–182 (Fig. 1B), that was previously described to interact with SOX9 and MYOCD [24]. For two variants, homozygotes are listed in gnomAD v4.1.0, total population (c.172A>G p.(Ser58Gly): 5 in 806,057 individuals, c.193G>A p.(Ala65Thr): 4 in 805,759 individuals), not unexpected in a condition, such as CAKUT, that may be unilateral (in 4 of 8 CAKUT patients carrying the c.172A>G p.(Ser58Gly) variant) and without impact on life expectancy, and that only affects fertility if combined with certain genital anomalies (in 2–3 of 8 CAKUT patients carrying the c.172A>G p.(Ser58Gly) variant) (Table 1). According to the ACMG/AMP guidelines [28], two variants were classified as likely pathogenic, i.e., c.172A>G p.(Ser58Gly) and c.188C>T p.(Pro63Leu), and three variants are of uncertain significance, i.e., c.193G>A p.(Ala65Thr), c.1879A>G p.(Lys627Glu), and c.2294C>T p.(Thr765Ile) (Table 1). Taken together, five different rare *TSHZ3* missense variants were detected in 12 CAKUT patients from 9 of 301 (3%) families.

### Hydronephrosis, hydroureter, and MCDK were the CAKUT phenotypes most specifically associated with rare *TSHZ3* variants

The phenotypes determined in the 12 CAKUT patients with rare *TSHZ3* variants by reverse phenotyping are depicted in Fig. 2A–K and summarized in Table 1 and Fig. 2L. Hydronephrosis and hydroureter were (significantly) more common in *TSHZ3* variant carriers compared to non-carriers (6/12 (50%) versus 68/301 (23%), *p* = 0.039, and 4/12 (33%) versus 39/301 (13%), *p* = 0.067, two-tailed Fisher’s exact test, Fig. 2L). Similarly, MCDK was more frequent in CAKUT patients with versus without *TSHZ3* variants (3/12 (25%) versus 21/301 (7%), *p* = 0.055, two-tailed Fisher’s exact test, Fig. 2L). The other anomalies in *TSHZ3* variant carriers, i.e., kidney (hypo)dysplasia, VUR, ureteropelvic junction obstruction, duplex kidney, posterior urethral valves, kidney cysts/cystic kidney dysplasia, and horseshoe kidney, were not present significantly more frequently than in non-carriers (Fig. 2L).

A literature search of publications reporting patients with heterozygous deletions at 19q12-q13.1 encompassing the *TSHZ3* locus revealed CAKUT phenotypes in 5/14 cases, i.e., hydronephrosis in 2/5 cases, small echogenic kidneys, hydroureter, or VUR grade II in 1/5 cases each [31–35].

### Genital anomalies and developmental delay were the extrarenal features associated with rare *TSHZ3* variants

Congenital extrarenal anomalies were observed in 9/12 (75%) patients carrying rare *TSHZ3* variants (Table 1). Among these features, genital anomalies and developmental delay were significantly more frequent in CAKUT patients with versus without rare *TSHZ3* variants (5/12 (42%) versus 44/291 (15%), *p* = 0.029, and 3/12 (25%) versus 17/291 (6%), *p* = 0.037, respectively, two-tailed Fisher’s exact test). The genital anomalies recurrently observed in *TSHZ3* variant carriers were cryptorchidism and phimosis (Table 1). No data on extrarenal features were available for 10 CAKUT patients.

### The SM layer was disorganized in the renal pelvis and thin in the proximal ureter of the nephrectomy specimen of a *TSHZ3* variant carrier

**Fig. 3:**
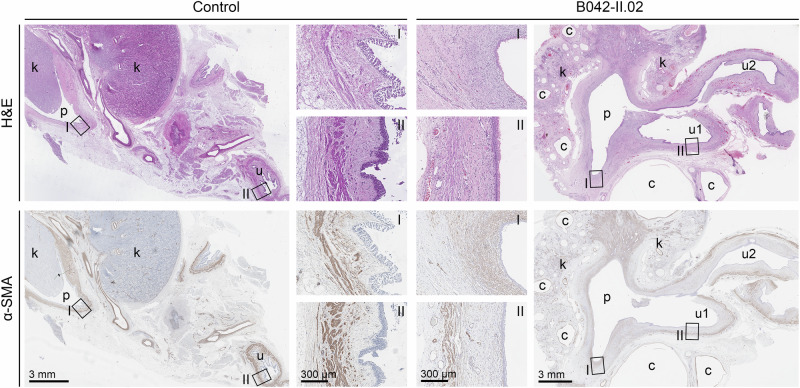
Histological characterization of a nephrectomy specimen from two-months-old male patient B042-II.02 (right panels) carrying the *TSHZ3*:c.172A>G p.(Ser58Gly) variant identified in five families, and a control specimen removed at autopsy from a one-year-old male with normal kidneys (left panels). Upper panels: H&E staining of the nephrectomy specimen of patient B042-II.02 revealed large cysts in the cortex and medulla of the hypodysplastic kidney without normal tissue, a dilated pelvicalyceal space, and a splitting of the proximal dilated ureter into two parts (u1 and u2) leading to the diagnoses MCDK, hydronephrosis, and duplex hydroureter. Compared to the control, the urothelium was thinner in the kidney pelvis (inset I) and the proximal ureter (inset II) of patient B042-II.02. Lower panels: immunostaining of the SM marker α-SMA. Compared to the control, α-SMA expression was diffuse and unstructured in the wall of the kidney pelvis (inset I), and reduced in the proximal ureter (inset II) of the nephrectomy specimen of patient B042-II.02, suggesting that the development of the SM layer is impaired due to the *TSHZ3* variant. Scale bars are as indicated. c cyst, k kidney, p pelvis, u ureter.

The nephrectomy specimen of male patient B042-II.02 carrying the *TSHZ3*:c.172A>G p.(Ser58Gly) variant, which was observed in a total of five patients, was available for further characterization. Hematoxylin and eosin (H&E) staining showed (i) a small dysplastic kidney without normal kidney tissue, and with large cysts in the cortex and medulla, (ii) a dilatation of the pelvicalyceal space, and (iii) a splitting of the dilated proximal ureter into two segments, consistent with the diagnoses MCDK, hydronephrosis, and duplex hydroureter (Fig. 3, Table 1). Since dilated thin-walled proximal ureters with no or reduced immunostaining for SM marker aortic smooth muscle actin (α-SMA) have been observed in *Tshz3*-null mutant mice at E17.5 [20], we investigated α-SMA expression in the nephrectomy specimen of the *TSHZ3* variant carrier by immunohistochemistry. Comparing the dysplastic kidney with a dilated pelvis of the *TSHZ3* variant carrier (patient B042-II.02) with an age- and sex-matched normal kidney (Fig. 3), and with the dysplastic kidney and dilated pelvis of a male pediatric patient without a rare *TSHZ3* variant (Supplementary Fig. 1), α-SMA staining in the wall of the kidney pelvis was diffuse and unstructured in the *TSHZ3* variant carrier (Fig. 3, Supplementary Fig. 1), suggesting that the differentiation of SM cells is impaired due to the *TSHZ3* variant. Similarly, in the proximal ureter, the SM layer was thinner in patient B042-II.02 carrying the *TSHZ3* variant than in the normal kidney (Fig. 3).

### *TSHZ3* is expressed in the human fetal brain and kidney, and in the developing murine brain, kidney, ureter, and bladder, among other organs

**Fig. 4:**
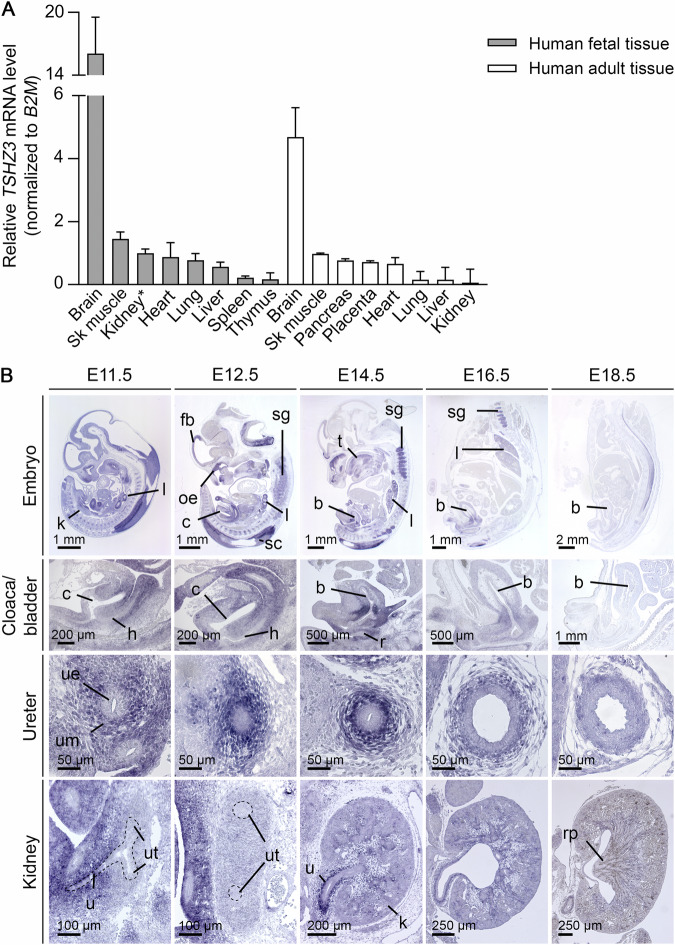
*TSHZ3* expression in human and murine tissues. **A** *TSHZ3* mRNA expression was quantified in cDNA extracted from multiple human fetal and adult tissues by real-time PCR, normalized to *B2M* mRNA, and displayed relative to mRNA levels in the fetal kidney (marked by an asterisk). *TSHZ3* mRNA expression was highest in the human fetal brain, followed by skeletal muscle and kidney compared to other fetal tissues analyzed. Data shown are mean ± standard deviation (error bars) from three independent experiments performed in triplicate. **B** *Tshz3* expression pattern by RNA in situ hybridization on sagittal sections of whole murine embryos and murine bladder, transverse sections of the ureter, and sagittal sections of the kidneys from E11.5 to E18.5. *Tshz3* is strongly expressed in the murine spinal cord and mesenchymal compartments of visceral tubular organs including the ureter from E11.5 to E14.5. Expression in the murine kidneys was confined to the medullary stroma. For each embryonic stage and organ, at least three specimens were analyzed. Scale bars are as indicated. b bladder, c cloaca, fb forebrain, h hindgut, k kidney, l lung, oe olfactory epithelium, r rectum, rp renal papilla, sc spinal cord, sg spinal ganglia, t tongue, u ureter, ue ureteric epithelium, um ureteric mesenchyme, ut ureter tips.

To explore whether *TSHZ3* is expressed during development in tissues affected in patients with *TSHZ3* variants, we quantified *TSHZ3* mRNA levels using cDNA panels from various human fetal and adult tissues. *TSHZ3* expression was highest in human fetal brain, skeletal muscle, and kidney (Fig. 4A). In the human fetal kidney, relative *TSHZ3* mRNA levels were about 17 times higher than in human adult kidney (Fig. 4A). The expression pattern of *Tshz3* during murine development was determined in wild-type mouse embryos using RNA in situ hybridization on sections of whole embryos, trunks, or urogenital systems at E11.5, E12.5, E14.5, E16.5, and E18.5 (Fig. 4B). At E11.5 and E12.5, strong *Tshz3* expression occurred in the developing fore- and hindbrain. Strong expression extended until E14.5 in the spinal cord and the spinal ganglia, the olfactory epithelium, and the tongue muscle. Additional sites of expression at these stages included the mesenchymal compartments of the developing lung, ureter, bladder, and intestine. Expression in the kidneys was confined to the medullary stroma. Expression at all of these sites was diminished at E16.5 and further reduced at E18.5 (Fig. 4B).

### N-terminal TSHZ3 variants affect binding of SOX9 and MYOCD

**Fig. 5:**
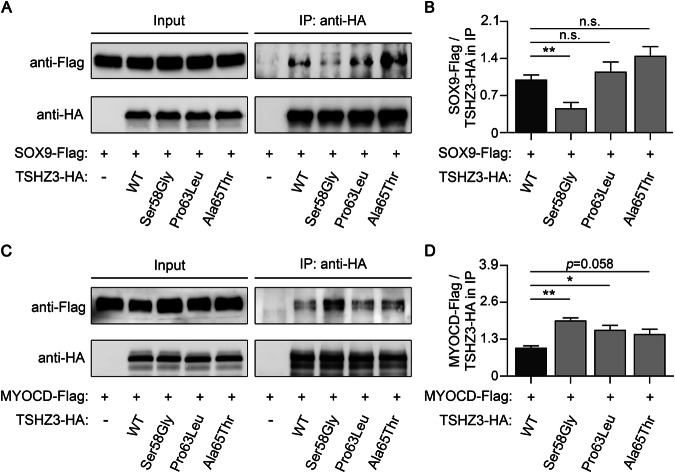
Characterization of TSHZ3-mutant proteins by co-immunoprecipitation (IP). **A**, **C** To explore the consequences of the three TSHZ3 variants located in the SOX9/MYOCD interaction region, binding of TSHZ3-HA wild-type or mutant (Ser58Gly, Pro63Leu, or Ala65Thr) proteins to either SOX9-Flag or MYOCD-Flag was analyzed by IP. After IP using anti-HA beads, Western blot analysis was performed to detect either SOX9-Flag (**A**) or MYOCD-Flag (**C**) and TSHZ3-HA in the IP eluate. **B**, **D** Densitometric measurement of protein bands revealed a significantly decreased ratio of SOX9-Flag to TSHZ3-HA Ser58Gly mutant (**B**), and a (significantly) increased ratio of MYOCD-Flag to all TSHZ3-HA mutants (**D**) compared to wild-type TSHZ3-HA in the immunoprecipitates, indicating that N-terminal TSHZ3 variants affect binding to SOX9 and/or MYOCD (data shown are mean ± standard deviation (error bars) from four or three independent experiments). *p < 0.05; **p < 0.01 (Student’s *t*-test).

Three *TSHZ3* missense variants, identified in 7/9 (78%) CAKUT families carrying a *TSHZ3* variant, affect amino acid residues in close proximity to each other that are conserved in species with a metanephric kidney, such as land vertebrates (e.g., reptiles, birds, and mammals) and salt water mammals (Supplementary Fig. 2). The three residues are located in an N-terminal region of TSHZ3 (Fig. 1B) reported to interact with the SOX9 and MYOCD transcription factors [24]. Rare (MAF < 0.002 according to gnomAD v4.1.0, total population) non-silent *TSHZ3* variants within this region (amino acids 1–182 of TSHZ3) were significantly more frequent in CAKUT patients compared to gnomAD samples (3.2% versus 0.7%, *p* = 0.000078447, two-tailed Fisher’s exact test). To explore the functional consequences of the three *TSHZ3* variants, we determined binding of SOX9 and MYOCD to wild-type and mutant (p.(Ser58Gly), p.(Pro63Leu), or p.(Ala65Thr)) TSHZ3 proteins in a co-immunoprecipitation assay (Fig. 5). While SOX9 binding was significantly impaired in the TSHZ3 p.(Ser58Gly) mutant (Fig. 5A, B), binding to MYOCD was (significantly) increased in all N-terminal TSHZ3 mutants compared to wild-type TSHZ3 (Fig. 5C, D). Considering the roles of TSHZ3, SOX9, and MYOCD in the initiation and progression of ureteral smooth muscle differentiation [24], both the reduced interaction between mutant TSHZ3 and SOX9, and the increased binding of mutant TSHZ3 to MYOCD may adversely affect smooth muscle differentiation.

## Discussion

Based on WES results in two siblings with unilateral MCDK, we report rare heterozygous missense variants in the *TSHZ3* gene in 12 patients from 9 of 301 (3%) CAKUT families, affecting an N-terminal region of the protein in most cases. These data provide evidence that the human *TSHZ3* gene, like its murine homolog, is associated with CAKUT. In line with the CAKUT phenotype observed in both siblings, MCDK but also hydronephrosis and hydroureter were more commonly observed in patients with versus without *TSHZ3* variants, defining quite a specific CAKUT phenotype spectrum for *TSHZ3* variant carriers. Specific extrarenal features, i.e., genital anomalies and developmental delay, were significantly more frequent in patients with versus without *TSHZ3* variants. Thereby, we establish the phenotype spectrum of CAKUT patients carrying rare heterozygous *TSHZ3* missense variants (Supplementary Fig. 3).

Teashirt (tsh) was initially described in *Drosophila* as a regulator of ventral trunk development with a spatial expression in the anteroposterior axis resembling a “T-shirt”, which led to the name [36, 37]. The mammalian *Tsh* gene family comprises the *Tshz1*, *Tshz2,* and *Tshz3* genes. *Tshz3* is expressed in SM cell precursors of the murine ureter [20]. Null mutations of *Tshz3* in mice lead to disorganized mesenchymal cells and missing expression of the SM marker α-SMA in the proximal ureter, defective ureteric contractility, and a prominent proximal hydroureter and hydronephrosis, a fully penetrant bilateral phenotype affecting both sexes, reminiscent of human congenital ureteropelvic junction obstruction [20] (Supplementary Fig. 3). Heterozygous *Tshz3*-mutant mice show decreased viability and unilateral hydroureter in 5–27% of cases [20, 35, 38]. Furthermore, glomerular density and glomerular basement membrane thickness were reduced in adult heterozygous *Tshz3*-mutant mice [21]. These findings are in line with in utero experimental data that decreased glomerular number may be a consequence of urinary tract obstruction during nephrogenesis [39], although in *Tshz3*-mutant mice a functional urinary flow impairment was observed. Overlapping with the phenotype in *Tshz3*-mutant mice, we observed hydronephrosis, hydroureter, ureteropelvic junction obstruction, and MCDK (significantly) more frequently in heterozygous *TSHZ3* variant carriers versus non-carriers in our CAKUT cohort. Moreover, α-SMA immunohistochemistry of the MCDK of a patient with the heterozygous TSHZ3 p.(Ser58Gly) variant, which affects in vitro binding to SOX9 and MYOCD, revealed a disorganized or thinner SM layer in the hydronephrotic pelvis and the proximal hydroureter. Extrarenal anomalies in *Tshz3*-mutant mice include autism spectrum disorder (ASD)-like behavioral deficits and abnormal central respiratory rhythm generation [35, 38, 40, 41]. While developmental delay was significantly more frequent in heterozygous *TSHZ3* variant carriers versus non-carriers in our CAKUT cohort, ASD was not observed in CAKUT patients carrying *TSHZ3* missense variants. The overlap of the CAKUT spectrum in *Tshz3*-mutant mice and individuals with heterozygous *TSHZ3* missense variants reported here (Supplementary Fig. 3) suggests a conserved role of TSHZ3 during urogenital tract development in the murine and human context.

The CAKUT phenotypes detected in *Tshz3*-mutant mice from E16.5 [20, 35, 38] and most frequently in carriers of *TSHZ3* missense variants of this study, i.e., hydronephrosis, hydroureter and/or MCDK, may be linked to the expression pattern of *TSHZ3*. Using RNA in situ hybridization on wild-type mice, *Tshz3* expression was observed in the mesenchymal compartment of the ureter from E11.5 and in the medullary stroma of the kidney from E14.5, was diminished at E16.5, and further reduced at E18.5. By immunostaining, TSHZ3 expression was maintained in ureteric mesenchymal cells at E18.5 [20]. Recently, we reported quite a similar expression pattern of the *Dact1* (dishevelled binding antagonist of beta-catenin 1) gene encoding a cytoplasmic WNT signaling mediator associated with human [42] and murine CAKUT [43, 44]. Comparable to *Tshz3*, *Dact1* expression was found in the mesenchyme of the ureter at E11.5 and additionally in the stroma of the kidney at E12.5 and E14.5, was reduced at E16.5, and strongly diminished at E18.5 [42]. Furthermore, single-cell transcriptomics on human fetal kidney of 16 weeks of gestation detected a highly similar *TSHZ3* and *DACT1* expression pattern predominantly in interstitial (progenitor) cells [45]. The similarity in *TSHZ3* and *DACT1* expression in the kidney and ureter is paralleled by a comparable CAKUT phenotype in carriers of *TSHZ3* and *DACT1* variants, i.e., MCDK or cystic kidney dysplasia, hydronephrosis and/or hydroureter [42]. These data confirm that expression in the ureter mesenchyme may be associated with hydroureter and hydronephrosis formation, and additionally suggest that expression in the kidney stroma may be associated with MCDK development in humans, as observed in *TSHZ3* and *DACT1* variant carriers.

Most of the *TSHZ3* variants detected in our CAKUT patients were located within the SOX9 and MYOCD interaction region of TSHZ3 [24] and led to the altered binding of mutant TSHZ3 to SOX9 and/or MYOCD. In undifferentiated mesenchymal precursors of the ureter, TSHZ3 likely interacts with SOX9 to promote SM cell differentiation through the transcriptional activation of MYOCD [20, 24]. Consequently, the significantly reduced SOX9 binding of mutant TSHZ3 harboring the p.(Ser58Gly) variant that we identified here, may lead to a decreased transcription of *MYOCD* in undifferentiated SM cells resulting in the failure of these cells to differentiate. In differentiating SMCs, MYOCD regulates SM gene expression through an interaction with serum response factor (SRF) [46] (https://www.wikipathways.org/pathways/WP1991.html). Displacement of MYOCD from SRF can repress SM gene expression [46, 47]. As TSHZ3 competes with SRF for binding to MYOCD [24], the significantly increased MYOCD binding of the TSHZ3 mutants harboring the N-terminal variants detected here biochemically, may prolong MYOCD displacement from SRF in differentiating SM cells, prevent the activation of SM genes, and perturb SM cell differentiation during development. Thus, we provide in vitro evidence that the N-terminal TSHZ3 variants identified in CAKUT patients may adversely affect SM cell differentiation in the kidney pelvis and proximal ureter in vivo, as suggested by α-SMA immunohistochemistry on the nephrectomy specimen of a *TSHZ3* variant carrier. Interestingly, in vivo inactivation of *Sox9* or *Myocd* in mice also causes hydronephrosis/proximal hydroureter or megabladder due to disruption of SM cell differentiation during urogenital system development [48, 49].

The phenotype spectrum of CAKUT patients carrying heterozygous *TSHZ3* missense variants partially overlaps with that previously described in patients with heterozygous 19q12-q13.11 deletions encompassing the *TSHZ3* gene locus (Supplementary Fig. 3). In 14 patients with 19q12-q13.11 deletions including *TSHZ3*, the characteristic features were feeding difficulty, developmental delay including speech delay, cognitive impairment or intellectual disability, and autistic behavior [31–35]. In addition, genital anomalies or CAKUT, including small echogenic kidneys, hydronephrosis, and hydroureter, were noted in two or five patients, respectively, with 19q12-q13.11 deletions encompassing *TSHZ3* [31–35]. Overlapping phenotypes in CAKUT patients with *TSHZ3* missense variants and patients with deletions of 19q12-q13.11 encompassing *TSHZ3* thus include developmental delay, intellectual disability, genital anomalies, and CAKUT. Further evidence for a link between heterozygous *TSHZ3* aberrations in humans and CAKUT, particularly hydronephrosis and hydroureter, come from two studies reporting hydronephrosis and ureteropelvic junction obstruction in one patient with a rare *TSHZ3* missense variant, i.e., c.724G>C p.(Asp242His) [22], and speech delay, intellectual disability, behavioral issues, hydronephrosis, and mild urethral stenosis in a patient with a heterozygous *TSHZ3* frameshift variant, i.e., c.119_120dup p.(Pro41SerfsTer79) [23]. However, we observe incomplete penetrance of CAKUT and variable expressivity of extrarenal phenotypes in carriers of *TSHZ3* missense variants, who may have a normal urogenital tract and/or intellectual development. Similarly, only 5–27% of heterozygous *Tshz3*-mutant mice present with a hydronephrosis/proximal hydroureter phenotype [20, 35], and only 5 of 14 (36%) patients with 19q12-q13.11 deletions including the *TSHZ3* locus are affected by CAKUT [31–35]. In autosomal dominant familial CAKUT, variable expressivity, and incomplete penetrance are common, and risk factors other than genetic aberrations, e.g., epigenetic regulation or environmental interactions, may also contribute to CAKUT pathogenesis and severity of anomalies [9–11].

In summary, evidence that *TSHZ3* is associated with the development of CAKUT, particularly MCDK, hydronephrosis and proximal hydroureter, in humans and mice comes from (i) this study describing rare heterozygous *TSHZ3* missense variants in 12 CAKUT patients from 9 of 301 (3%) families, (ii) previous reports of rare *TSHZ3* variants in two CAKUT patients [22, 23], (iii) previous reports of a CAKUT phenotype in five patients with heterozygous 19q12-q13.11 deletions encompassing *TSHZ3* [35], and (iv) the study of *Tshz3* mutant mice [20, 21]. We show here that N-terminal TSHZ3 variants affect binding of SOX9 and MYOCD, providing a pathomechanism by which *TSHZ3* missense variants may lead to disrupted SM cell differentiation, as suggested by α-SMA immunohistochemistry on a nephrectomy specimen of a *TSHZ3* variant carrier with MCDK, hydronephrosis and hydroureter, and previously shown in the proximal ureter of *Tshz3*-null mutant mice [20]. As genital anomalies and developmental delay were significantly more frequent in CAKUT patients with versus without rare *TSHZ3* variants, observing combined hydronephrosis/-ureter, genital anomalies and cognitive impairment in patients is suggestive of a *TSHZ3* aberration.

## Supplementary information

Supplementary material

## Data Availability

All relevant data are available in the manuscript or supplementary information.
