# Supplementary material for: Heterozygous variants in the teashirt zinc finger homeobox 3 (TSHZ3) gene in human congenital anomalies of the kidney and urinary tract
Source: Eur J Hum Genet. 2024 Oct 17;33(1):44–55. doi: 10.1038/s41431-024-01710-y (PMC11711546; doi:10.1038/s41431-024-01710-y)
Supplement: Supplementary file 1 — Supplementary material [file 41431_2024_1710_MOESM1_ESM.pdf]

Supplementary material

Heterozygous variants in the teashirt zinc finger homeobox 3 (*TSHZ3*) gene in human congenital anomalies of the kidney and urinary tract

Esra Kesdiren[1], Helge Martens[1], Frank Brand[1], Lina Werfel[1,2], Lukas Wedekind[3], Mark-Oliver Trowe[3], Jessica Schmitz[4], Imke Hennies[2], Robert Geffers[5], Zoran Gucev[6], Tomáš Seeman[7,8], Sonja Schmidt[9], Velibor Tasic[6], Laurent Fasano[10], Jan H. Bräsen[4], Andreas Kispert[3], Anne Christians[1], Dieter Haffner[2], Ruthild G. Weber[1]

[1]Department of Human Genetics, Hannover Medical School, Hannover, Germany;

[2]Department of Pediatric Kidney, Liver, Metabolic and Neurological Diseases, Hannover Medical School, Hannover, Germany;

[3]Institute of Molecular Biology, Hannover Medical School, Hannover, Germany;

[4]Nephropathology, Department of Pathology, Hannover Medical School, Hannover, Germany;

[5]Genome Analytics Research Group, Helmholtz Centre for Infection Research, Braunschweig, Germany;

[6]Pediatric Nephrology, University Children's Hospital, Skopje, North Macedonia;

[7]Department of Pediatrics, 2nd Faculty of Medicine, Charles University, Prague, Czech Republic;

[8]Department of Pediatrics, Faculty of Medicine, University of Ostrava, Czech Republic

[9]Department of General-, Visceral- and Pediatric Surgery, University Medical Center Göttingen, Göttingen, Germany

[10]Aix-Marseille Univ, CNRS, IBDM, UMR7288 Marseille, France

Correspondence to: Ruthild G. Weber, Department of Human Genetics OE 6300, Hannover Medical School, Carl-Neuberg-Str. 1, 30625 Hannover, Germany, Phone: +49 511 5327751, Fax: +49 511 53218520, E-mail: weber.ruthild@mh-hannover.de

D. Haffner and R. G. Weber contributed equally as senior authors

SUPPLEMENTARY MATERIALS AND METHODS

Case reports of patients carrying *TSHZ3* variants

Rare heterozygous *TSHZ3* missense variants predicted to be deleterious were identified in 12 CAKUT patients (F004-II.02, F004-II.03; B016-II.02, B016-I.02; B042-II.02; C012-II.01; H422-II.02, H422-I.02; A025-II.01; CZE006-II.02; H317-II.02; GOE003-II.01) from 9 of 301 families. Information on patients F004-II.02 and F004-II.03 of the index family is provided in the results section of the main document.

B016-II.02 and B016-I.02: NM_020856.4(*TSHZ3*):c.172A>G p.(Ser58Gly)

Patient B016-II.02, a girl, is the second liveborn child of non-consanguineous Turkish parents, and was born prematurely at 29+0 weeks gestational age by Cesarean section. During pregnancy, maternal diabetes was diagnosed. At birth, weight was 825 g (7th percentile), body length was 37 cm (30th percentile), and head circumference was 25 cm (15th percentile). After birth, the girl was diagnosed with persistent ductus arteriosus, atrial septal defect, and hemangioma. In addition, she presented with respiratory distress syndrome, retinopathy of prematurity, and neonatal hyperbilirubinemia. Kidney ultrasonography was notable for bilateral kidney hypodysplasia (Fig. 2C). Kidney function was impaired. At the age of 8 1/12 years, the girl received preemptive kidney transplantation (KTx), but unfortunately showed a borderline rejection after three years. At 14 years of age, kidney function was classified as chronic kidney disease (CKD) stage 4 with arterial hypertension, renal acidosis, and renal anemia. Her mother, patient B016-I.02, who had one spontaneous abortion also carried the *TSHZ3* variant. Kidney ultrasonography revealed bilateral kidney cysts. An ovarian cyst was also observed. The two brothers in whom *TSHZ3* variant status could not be determined were clinically unremarkable. No clinical information was available on the father.

B042-II.02: NM_020856.4(*TSHZ3*):c.172A>G p.(Ser58Gly)

Patient B042-II.02, a boy, is the second liveborn child of non-consanguineous parents. Due to maternal indication, birth was induced at 36+3 weeks gestational age by Cesarean section.

The fetus was prenatally diagnosed with oligohydramnios and bilateral hydronephrosis. After birth, peritoneal dialysis was started because of anuria. A kidney ultrasound revealed right-sided MCDK and bilateral hydronephrosis (Fig. 2D). Voiding cystourethrogram showed posterior urethral valves (PUV) with bilateral high-grade vesicoureteral reflux (VUR) and bilateral hydroureter (Fig. 2D). Right-sided urethrectomy and nephrectomy was performed at the age of four weeks. Macroscopic examination of the nephrectomy specimen showed numerous cysts (maximum diameter 0.8 cm) on the surface and lamellar cuts of the kidney. The proximal ureter was dilated and divided into two parts joining to form one ureter 3.1 cm distal from the renal pelvis, no stenoses were noted. Microscopic examination revealed kidney parenchyma with cystic transformation, primitive immature tubules and glomeruli, and interstitial hyalinized fibrosis. Cysts were lined by cuboidal epithelium. The findings correspond to the clinical diagnosis of multicystic dysplastic kidney and dilated, partially duplex ureter. At the age of four months, primary transurethral valve ablation was performed. Continuous deterioration of kidney function up to CKD stage 4 was observed over 8 years. As a consequence of CKD, the patient developed short stature and secondary hyperparathyroidism. He was listed for pre-emptive KTx**.** The patient also presented with pulmonary hypoplasia, muscle hypotonia, hypoxic ischemic encephalopathy, developmental delay, and epilepsy. Other anomalies were microcephaly, bilateral pes calcaneus, and neurogenic bladder dysfunction. Bilateral cryptorchidism was surgically treated at the age of 7 years. His parents and sister were not available for kidney ultrasonography or genetic testing. His mother had three spontaneous abortions.

C012-II.01: NM_020856.4(*TSHZ3*):c.172A>G p.(Ser58Gly)

Patient C012-II.01, a boy, is the only child of non-consanguineous German parents. At the age of 4 weeks, left-sided hydronephrosis was diagnosed by kidney ultrasonography (Fig. 2E). At the age of two months, he was affected by urosepsis. Voiding cystourethrogram and kidney scintigraphy revealed left-sided hydroureter and bilateral VUR without relevant stenosis. At the age of six months, the patient was again affected by urosepsis despite antibiotic prophylaxis

since the first urosepsis. Except for phimosis, no other anomalies were noted. No kidney ultrasonography was done in his parents.

H422-II.02 and H422-I.02: NM_020856.4(*TSHZ3*):c.172A>G p.(Ser58Gly)

Patient H422-II.02, a boy, is the second child of non-consanguineous North Macedonian parents. Kidney ultrasonography revealed left-sided hydronephrosis and ureteropelvic junction obstruction (Fig. 2F). Additionally, he presented with bilateral clinodactyly of the fourth and fifth toe. Kidney ultrasonography of his mother, patient H422-I.02, also carrying the *TSHZ3* variant, showed right-sided non-obstructive duplex kidney (Fig. 2G). Ultrasonography of the kidney and bladder of the patient's father and sister were unremarkable.

A025-II.01: NM_020856.4(*TSHZ3*):c.188C>T p.(Pro63Leu)

Patient A025-II.01, a boy, is the first child of non-consanguineous German parents born after an unremarkable pregnancy. Ultrasound screening after birth showed bilateral hydronephrosis. Diagnostic work-up revealed bilateral small hyperechogenic kidneys with reduced parenchymal thickness and corticomedullary differentiation, hydronephrosis and hydroureters. Voiding cystourethrogram revealed bilateral VUR grade IV (Fig. 2H). Cystoscopy showed gaping ureter ostia, and excluded PUV. Based on these findings, bilateral kidney hypodysplasia, hydronephrosis, hydroureter combined with grade IV VUR were diagnosed. The patient showed progressive CKD requiring start of peritoneal dialysis at the age of 4 years. At the age of five years, he had severe pyelonephritis. He underwent right-sided urethrectomy and nephrectomy, and KTx at the age of 5 8/12 years. Macroscopic examination of the nephrectomy specimen confirmed loss of normal corticomedullary differentiation and a dilated kidney pelvis. Microscopic examination revealed hypoplasia of kidney tissue and chronic interstitial nephritis due to hydroureters and chronically recurrent inflammation. Additionally, the boy was diagnosed with developmental delay and left-sided retractile testis. He also presented with short stature due to growth hormone deficiency and CKD. His parents were not available for kidney ultrasonography.

CZE006-II.02: NM_020856.4(*TSHZ3*):c.193G>A p.(Ala65Thr)

Patient CZE006-II.02, a boy, is the second child of a Czech couple. Prenatal kidney ultrasonography revealed bilateral hydronephrosis. Postnatal kidney ultrasonography confirmed bilateral hydronephrosis together with bilateral hydroureter. By voiding cystourethrogram, bilateral VUR grade IV-V together with posterior urethral valves were diagnosed (Fig. 2I). The posterior urethral valves were dissolved by cystoscopy. Follow-up kidney ultrasonography diagnosed bilateral reflux nephropathy. The boy progressed slowly into end-stage CKD, started chronic hemodialysis at the age of 7 years and underwent KTx at the age of 9 years.

H317-II.02: NM_020856.4(*TSHZ3*):c.1879A>G p.(Lys627Glu)

Patient H317-II.02, a girl, is the second child of non-consanguineous North Macedonian parents born at term. Evaluation by dimercaptosuccinic acid kidney scan and kidney ultrasonography showed a horseshoe kidney without signs of dysplasia (Fig. 2J). Additionally, high-arched palate, right-sided overfolded ear helix, and left-sided single transverse palmar crease were diagnosed. Ultrasonography of the kidney and bladder of her parents and her brother was unremarkable.

GOE003-II.01: NM_020856.4(*TSHZ3*):c.2294C>T p.(Thr765Ile)

Patient GOE003-II.01, a boy, is a child of non-consanguineous German parents and has three siblings. Kidney ultrasonography and magnetic resonance imaging revealed left-sided duplex kidney with dysplasia of the lower pole, hydronephrosis, and ureteropelvic junction obstruction (Fig. 2K). Kidney scintigraphy showed reduced function of the left-sided kidney, and no function of the lower pole of the duplex kidney. At the age of 12 years, a left-sided nephrectomy was performed. The patient was also diagnosed with developmental delay, dilated lateral ventricles, mega cisterna magna, and a pineal gland cyst. He is attending a school for children with special needs. Additionally, he presented with bilateral pes planus. His parents were not available for kidney ultrasonography.

Immunohistochemistry on human kidney specimens

Immunohistochemical analysis was performed on 3 µm sections of (i) the formalin-fixed paraffin-embedded MCDK from male patient B042-II.02, who carried a *TSHZ3* variant, that was surgically resected during kidney transplantation at 4 weeks of age, and of (ii) a normal human kidney and ureter from a one-year-old male infant removed at autopsy. Sections were stained with hematoxylin and eosin according to a standard protocol, or incubated with the monoclonal mouse antibody raised against human aortic smooth muscle actin (α-SMA) (clone 1A4, #760-2833, Roche, Basel, Switzerland). Immunostaining was done in a routine manner using an automated tissue staining system (VENTANA BenchMark ULTRA, Roche) and documented using the Aperio AT2 scanner (Leica Microsystems, Wetzlar, Germany). Images were analyzed using the ImageScope v12.3.3.5048 software (Leica Microsystems).

Quantitative analysis of *TSHZ3* mRNA expression in human fetal and adult tissue

To quantify *TSHZ3* mRNA expression in human fetal and adult tissues, the TaqMan Universal PCR Master Mix (#4304437, Thermo Fisher Scientific, Waltham, MA, USA) and the TaqMan Gene Expression Assay for *TSHZ3* (Hs01583885_m1, #4331182, Thermo Fisher Scientific) were used on Human Fetal MTC Panel (#636747), and Human MTC Panel I (#636742, both Takara Bio, Kusatsu, Japan). Each sample was normalized to TaqMan control target beta-2-microglobulin (*B2M*, Hs00187842_m1, Thermo Fisher Scientific), and comparative C_t quantification was applied.

RNA *in situ* hybridization on sections of murine embryos or kidneys during development

To determine the expression pattern of *Tshz3* during murine development, non-radioactive RNA *in situ* hybridization was carried out following a standard protocol (Moorman et al. 2011). In brief, embryos or urogenital systems of wildtype mice (ZtmHan:NMRI) fixed in 4% paraformaldehyde (PFA) were paraffin-embedded and sectioned to 10 µm thickness. Sections were deparaffinized in Carl Roth Roti-Histol (#10379029; Thermo Fisher Scientific),

sequentially rehydrated in ethanol/H_2O, washed in PBS, and treated with 10 µg/ml proteinase K (#7528; Carl Roth, Karlsruhe, Germany) in 0.1 M Tris, pH 8.0 at 37°C for 8 min. After washing with 0.2% glycerin/PBS and PBS, and post-fixation with 4% PFA/0.2% glutaraldehyde at room temperature (RT) for 20 min each, sections were hybridized with a digoxygenin-labeled riboprobe (DIG RNA Labeling Mix, #11277073910; Sigma-Aldrich, St. Louis, MO, USA) directed against mouse *Tshz3* mRNA in hybridization buffer at 70°C overnight. Sections were washed twice in 50% formamide/50% 2x saline-sodium citrate buffer (pH 7.0) at 65°C for 20 min. Probes were detected using Anti-Digoxigenin-AP, Fab fragments from sheep (2 h at RT) and BM Purple AP substrate (#11093274910 and #11442074001; Sigma-Aldrich). Stained sections were documented on a Leica DM5000 microscope using a Leica DFC300 FX digital camera (Leica Microsystems). For each developmental stage, at least three specimens were analyzed.

Cloning of *TSHZ3, MYOCD* and *SOX9* expression constructs and site-directed mutagenesis

To generate expression constructs, the full-length open reading frames of *TSHZ3*, *MYOCD,* and *SOX9* were amplified from human cDNA (obtained from Human Fetal MTC panel #636747, Takara Bio) and subcloned into the *pcDNA3.1-HA* vector (Thermo Fisher Scientific) or the *pCMV6-Entry-Myc-FLAG* vector (Origene, Rockville, MD, USA) using customized oligonucleotides (Supplementary Table 3) and the In-Fusion HD Cloning Kit (Takara Bio). Variants were inserted into the *TSHZ3* expression construct using customized oligonucleotides (Supplementary Table 3) and the Phusion Site-Directed Mutagenesis kit (Thermo Fisher Scientific). The generated constructs were analyzed by restriction digest, and inserted sequences and variants were verified using customized oligonucleotides (Supplementary Table 3) and conventional chain termination protocols.

Cell culture and transient transfection

Human embryonic kidney 293T (HEK293T) cells were cultured in high-glucose Dulbecco's Modified Eagle Medium (Merck, Darmstadt, Germany) supplemented with 10% fetal bovine serum, 100 units/ml penicillin, and 100 µg/ml streptomycin (all Thermo Fisher Scientific). Cell cultures were maintained at 37°C in a humidified atmosphere containing 5% CO_2. For transient transfection of HEK293T cells, Lipofectamine 3000 transfection reagent (Thermo Fisher Scientific) was used following standard protocols.

Immunoprecipitation

To analyze binding of wildtype and mutant TSHZ3-HA to SOX9-Myc-FLAG or MYOCD-Myc-FLAG by immunoprecipitation (IP), HEK293T cells ($1.0x10^7$) were transiently co-transfected with *pcDNA3.1-TSHZ3-HA* (wildtype or mutant) and *pCMV6-Entry-SOX9-Myc-FLAG* or *pCMV6-Entry-MYOCD-Myc-FLAG* 24 h after seeding. At 4 h after transfection, 1 µM proteasome inhibitor MG-132 (#474790, Merck) was added to the culture medium, and at 24 h after transfection, cells were lysed in IP buffer (50 mM Tris-HCl, pH 8.0, 50 mM sodium fluoride, 1 mM sodium orthovanadate, 1% Triton X-100) supplemented with protease and phosphatase inhibitors (#04693159001 and #4906837001, both Roche). Pierce Anti-HA Magnetic Beads (#88836, Thermo Fisher Scientific) equilibrated in IP buffer were incubated with the lysates overnight at 4°C. After washing with IP buffer, proteins were eluted from the beads using 2x Laemmli buffer (125 mM Tris-HCl, pH 6.8, 20% glycerol, 10% 2-mercaptoethanol, 4% sodium dodecyl sulfate (SDS), 2 mM ethylenediaminetetraacetic acid, 0.005% bromophenol blue) and detected by Western blot analysis.

Western blot analysis

After SDS-polyacrylamide gel electrophoresis and semidry electroblotting, nitrocellulose membranes (General Electric, Boston, MA, USA) were immersed in 5% fat-free milk powder dissolved in PBS with 0.05% Tween 20 (PBST) as blocking agent. Rabbit anti-HA-TAG (#3724, Cell Signaling Technology, Danvers, MA, USA) or mouse anti-FLAG-Tag (#8146, Cell

Signaling Technology) primary monoclonal antibodies were diluted at the ratio of 1:1,000 in 5% (w/v) bovine serum albumin in PBST and used for immunodetection. After incubation overnight at 4°C, the membranes were exposed to the secondary horseradish peroxidase-conjugated anti-rabbit antibody (1:3,000; #A16035, Thermo Fisher Scientific) or anti-mouse antibody (1:3,000; #A16017, Thermo Fisher Scientific) in 5% fat-free milk powder dissolved in PBST (1.5 h at RT), and developed using the SuperSignal West Dura Extended Duration Substrate (Thermo Fisher Scientific). For image acquisition, the Fusion FX7 gel documentation system (Vilber, Collégien, France) was used. Densitometric quantification of protein bands was performed using ImageJ software (Schneider et al. 2012).

SUPPLEMENTARY TABLES

Supplementary Table 1 Spectrum of kidney and/or urinary tract anomalies in 313 CAKUT patients from 301 families included in this study

CAKUT phenotype	Frequency
Bilateral kidney (hypo)dysplasia	81/313
- and VUR	30/81
- and PUV	10/81
- and PUV and hydroureter	5/81
- and hydronephrosis	4/81
- and PUV, hydroureter and hydronephrosis	3/81
- and UVJO	2/81
- and UVJO and hydronephrosis	2/81
- and ectopia	1/81
- and hydroureter	1/81
- and hydroureter and hydronephrosis	1/81
Unilateral kidney (hypo)dysplasia	49/313
- and VUR	17/49
- and hydronephrosis	7/49
- and PUV	5/49
- and ectopia	3/49
- and UPJO and hydronephrosis	1/49
- and UVJO	1/49
- and hydroureter	1/49
Unilateral kidney agenesis	29/313
- and contralateral kidney dysplasia	9/29
- and contralateral VUR	6/29
- and contralateral cystic kidney dysplasia	3/29
- and contralateral MCDK	1/29
- and contralateral UPJO and hydronephrosis	1/29
- and contralateral hydroureter	1/29
- and PUV	1/29
Bilateral cystic kidney dysplasia	28/313
- and VUR	6/28
- and hydronephrosis	2/28
- and PUV	2/28
- and hydroureter	1/28
Unilateral MCDK	24/313
- and VUR	2/24
- and contralateral cystic kidney dysplasia	1/24
- and contralateral kidney hypoplasia	1/24
- and contralateral UPJO and hydronephrosis	1/24
- and PUV, hydroureter and hydronephrosis	1/24
Unilateral UPJO	22/313
- and ipsilateral hydronephrosis	16/22
Unilateral duplex kidney	18/313
- and hydronephrosis	5/18
- and hydroureter	4/18
- and VUR	3/18
- and UVJO	2/18
- and hydroureter and hydronephrosis	2/18
- and bilateral kidney dysplasia	2/18
- and contralateral kidney triplication	1/18
- and contralateral MCDK	1/18
- and contralateral kidney dysplasia	1/18

- and ipsilateral dysplasia and UPJO<br>- and ureteric ectopia<br>- and UPJO	1/18<br>1/18<br>1/18
Unilateral cystic kidney dysplasia<br>- and ectopia<br>- and kidney hypoplasia and VUR	10/313<br>1/10<br>1/10
Unilateral hydronephrosis<br>- and VUR	10/313<br>3/10
Crossed fused renal ectopia<br>- and VUR<br>- and hydroureter and hydronephrosis<br>- and ipsilateral kidney dysplasia	8/313<br>3/8<br>2/8<br>1/8
PUV<br>- and VUR<br>- and hydroureter and hydronephrosis<br>- and hydronephrosis	6/313<br>3/6<br>2/6<br>1/6
Horseshoe kidney<br>- and hydroureter	6/313<br>1/6
Bilateral hydronephrosis<br>- and VUR	5/313<br>1/5
Unilateral UVJO<br>- and hydronephrosis<br>- and VUR	5/313<br>3/5<br>1/5
Bilateral duplex kidney<br>- and kidney dysplasia, duplex ureter and PUV<br>- and VUR	4/313<br>1/4<br>1/4
Bilateral UVJO and hydronephrosis	3/313
Pelvic kidney	2/313
Bilateral hydroureter<br>- and bilateral hydronephrosis and VUR<br>- and VUR	2/313<br>1/2<br>1/2
Unilateral hydroureter and hydronephrosis	1/313

MCDK, multicystic dysplastic kidney; PUV, posterior urethral valves; UPJO, ureteropelvic junction obstruction; UVJO, ureterovesical junction obstruction; VUR, vesicoureteral reflux

Supplementary Table 2 Linkage- and candidate-based analysis of WES data from affected siblings F004-II.02 and F004-II.03 identified a rare heterozygous *TSHZ3* missense variant

**Prioritization steps**	**Number of variants**
Total variants in exome obtained from leukocyte DNA	F004-II.02: 74,336<br>F004-II.03: 73,646
Linkage-based strategy: variants with a call quality of ≥50, read depth of ≥20 and an allele fraction of ≥45%, shared by CAKUT patients II.02 and II.03 of family F004 are retained	21,132
Non-silent variants, i.e. splice site (up to 2 bases into intron), frameshift, in-frame indels, stop gained/lost, and non-synonymous missense variants, are retained	3,815
Population filtering: rare variants (minor allele frequency ≤0.002 in the 1000 Genomes Project, ExAC database, gnomAD database v2.1.1 total population, or NHLBI ESP exomes)[a] are retained	123
Comparison with exome data of unrelated adults without clinical signs of impaired kidney health serving as controls (n=153) whose DNA was sequenced in parallel with that of CAKUT patients, variants not present in controls are retained	23
Variants predicted to be deleterious by at least one prediction program (MutationTaster, SIFT, PROVEAN or PolyPhen-2) are retained	23
Candidate-based strategy: variants in (candidate) genes associated with human isolated or syndromic CAKUT according to our in-house gene list (n=279 genes[b]) are retained	0
Candidate-based strategy: variants in genes associated with murine CAKUT according to our in-house gene list (n=170 genes[c]) are retained	1 (*TSHZ3*)

[a]According to QIAGEN Clinical Insight Interpret Translational: 1000 Genomes Project data (https://www.internationalgenome.org/), Exome Aggregation Consortium (ExAC; https://gnomad.broadinstitute.org/), Genome Aggregation Database (gnomAD; https://gnomad.broadinstitute.org/), NHLBI Exome Sequencing Project (NHLBI ESP, https://evs.gs.washington.edu/EVS/)

[b]Based on Nicolaou et al. 2016; Vivante and Hildebrandt 2016; Bondeson et al. 2017; Heidet et al. 2017; Kosfeld et al. 2018; van der Ven et al. 2018a; van der Ven et al. 2018b; Blackburn et al. 2019; Connaughton et al. 2019; Dong et al. 2019; Fernandez-Prado et al. 2019; Jain and Chen 2019; Kitzler et al. 2019; Kolvenbach et al. 2019; Mann et al. 2019; Rawlins et al. 2019; Barrie et al. 2020; Connaughton et al. 2020; Szot et al. 2020; Yang et al. 2020; Fabretti et al. 2021

[c]Based on Nicolaou et al. 2016; Heidet et al. 2017; van der Ven et al. 2018a; van der Ven et al. 2018b; Jain and Chen 2019

Supplementary Table 3 Oligonucleotides used for different applications, as indicated

**Designation**	**Sequence (5' to 3') and possible 5' phosphorylation (5'-PHOS)**
Amplification and Sanger sequencing of human *TSHZ3* coding exons	
TSHZ3_Ex1_F1	GCA GCA GCA TCC TGC G
TSHZ3_Ex1_R1	GGA GTT ACT CAG TGC GGG C
TSHZ3_Ex2_F1	CTC TCT TCA TTC CAT CCT ACC TG
TSHZ3_Ex2_R1	GGT CTC ATG GTT GTC GTC G
TSHZ3_Ex2_F2	CTC TAT GGC TCC ATC TTC ACG
TSHZ3_Ex2_R2	CGC TTT CTC CTT GTC GAC TTC
TSHZ3_Ex2_F3	GCC TGT CAC ACC TAC CAT CAC
TSHZ3_Ex2_R3	GCT TCA CCA GCT CCT TGC
TSHZ3_Ex2_F4	CAT CAA GAT GGA GGC ATC C
TSHZ3_Ex2_R4	CCT GGA GGA TCA GGA GGT G
TSHZ3_Ex2_F5	CAT CTC CGA GAA GTC TGA CAT TG
TSHZ3_Ex2_R5	GTC CAG CCC AGA GTG ATT CTC
Generation of *pcDNA3.1-TSHZ3-HA*, *pCMV6-Entry-MYOCD-Myc-FLAG* and *pCMV6-Entry-SOX9-Myc-FLAG* expression constructs	
InFusion-hTSHZ3_F	AGG GAG ACC CAA GCT TAT GCC GAG GAG GAA GCA GC
InFusion-hTSHZ3_R	CAT GGT GGC GAA GCT TAA CTG CTT CTC TAA CTC AGA GAC
InFusion-hMYOCD_F	CTG GAT CCG GTA CCG AGG AGA TCT GCC GCC GCG ATC GCC ATG ACA CTC CTG GGG TCT GAG
InFusion-hMYOCD_R	TCT GCT CGA GCG GCC GCT TGT ACC ACT GCT GCA AGT G
InFusion-hSOX9 _F	CGT CGA CTG GAT CCG GTA CCA TGA ATC TCC TGG ACC CCT TCA
InFusion-hSOX9_R	TCT GCT CGA GCG GCC GCC AAG GTC GAG TGA GCT GTG TGT
Site-directed mutagenesis of the *pcDNA3.1-TSHZ3-HA* expression construct	
hTSHZ3-HA_Stop_F	CAG ATT ACG CTT GAT CCA CTA GTA ACG GCC
hTSHZ3-HA_Stop_R_Phos	GAA CAT CGT ATG GGT AAG CCA TGG TGG (5'-PHOS)
hTSHZ3_c172A>G_F	CTG CCC CGG CTA CCA GAA CTC C
hTSHZ3_c172A>G_R_Phos	GCC CTG GCG AGC TCC TTC TCC (5'-PHOS)
hTSHZ3_c188C>T_F	AAC TCC CTG GCC GCC GAG TTT TC
hTSHZ3_c188C>T_R_Phos	CTG GTA GCT GGG GCA GGC CC (5'-PHOS)
hTSHZ3_c193G>A_F_Phos	CAG ATT ACG CTT GAT CCA CTA GTA ACG GCC (5'-PHOS)
hTSHZ3_c193G>A_R	GAA CAT CGT ATG GGT AAG CCA TGG TGG

Ex, exon; F, forward; h, human; Phos, phosphorylation; R, reverse

SUPPLEMENTARY FIGURES

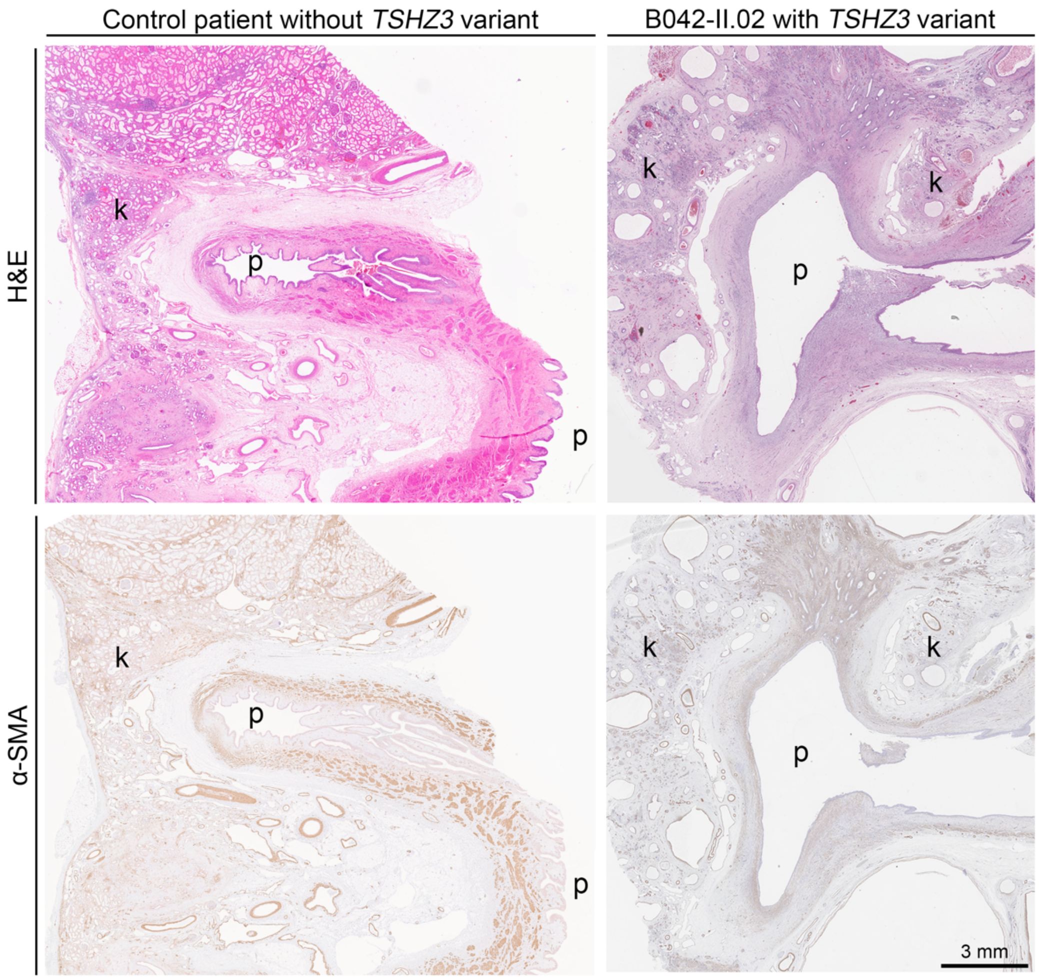

Supplementary Fig. 1 Histological comparison of dysplastic kidneys with a dilated pelvis in two male patients: patient B042-II.02 with a *TSHZ3*:c.172A>G p.(Ser58Gly) variant and a pediatric control patient without a rare *TSHZ3* variant. Upper panels: hematoxylin and eosin (H&E) staining. Lower panels: immunostaining of the smooth muscle marker α-SMA. Compared to the control dysplastic kidney, α-SMA expression was diffuse and unstructured in the pelvis wall of the dysplastic kidney of patient B042-II.02, suggesting an effect of the *TSHZ3* variant on the differentiation of smooth muscle cells in the kidney pelvis. The scale bar in the lower right panel applies to all panels. k, dysplastic kidney; p, pelvis

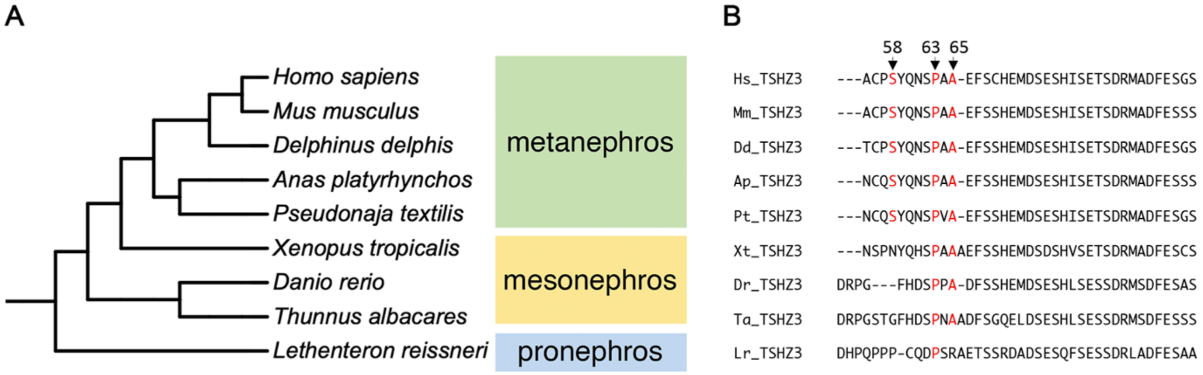

Supplementary Fig. 2 A Phylogenetic tree of selected species representative of animals with a pronephros (*Lethenteron reissneri* (lamprey)), a mesonephros (*Thunnus albacares* (tuna), salt water fish; *Danio rerio* (zebrafish), fresh water fish; *Xenopus tropicalis* (xenopus)), and a metanephros (*Pseudonaja textilis* (snake, reptile); *Anas platyrhynchos* (duck, bird); *Delphinus delphis* (dolphin), salt water mammal; *Mus musculus* (mouse) and *Homo sapiens* (human), terrestrial mammals). **B** Alignment of the TSHZ3 proteins from the selected species showing conservation of the amino acid residues S58, P63 and A65 (numbered for Hs_TSHZ3, marked in red and indicated by an arrow). Note that these three amino acid residues are conserved in species with a metanephros.

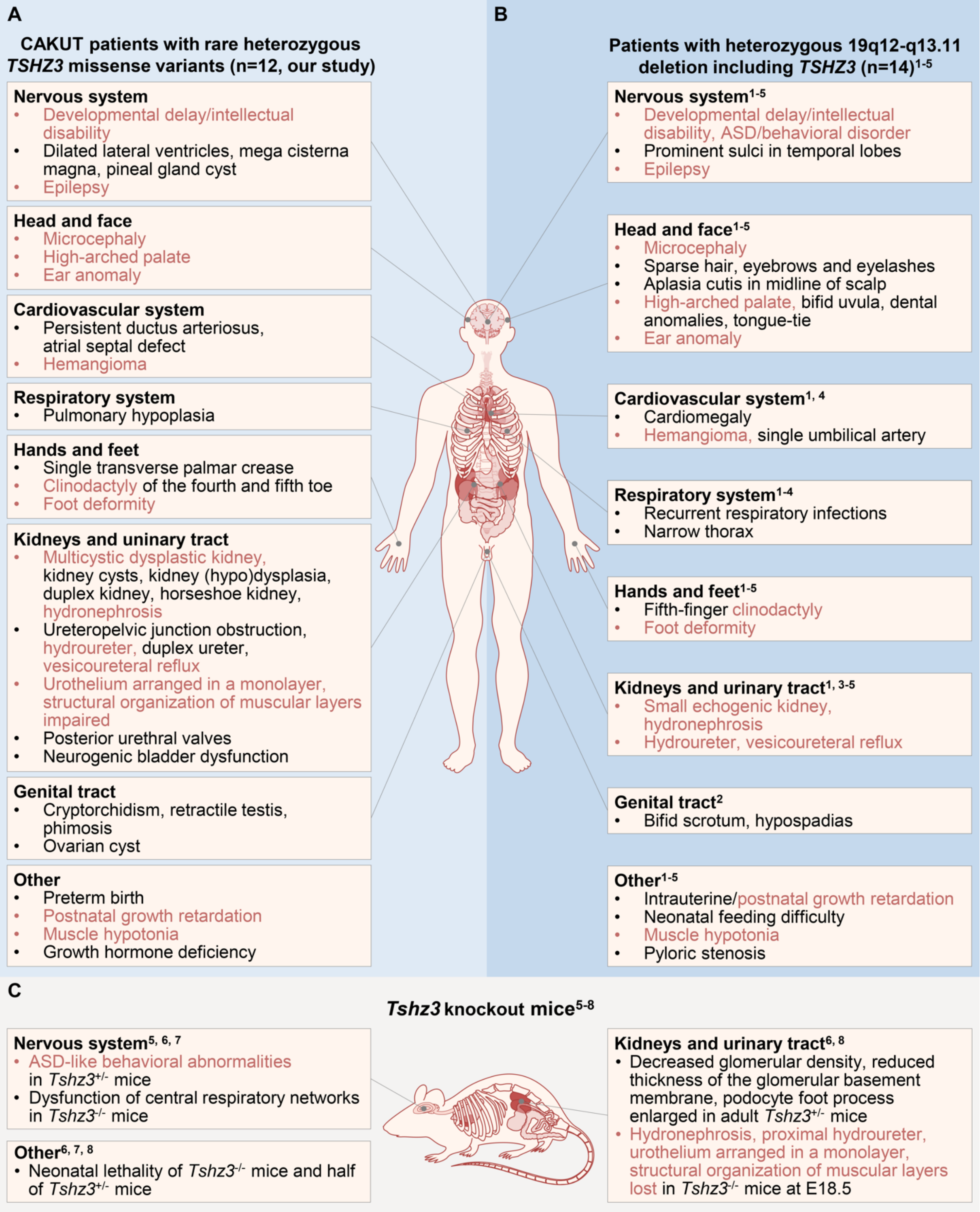

Supplementary Fig. 3 Comparison of features in CAKUT patients with rare heterozygous *TSHZ3* missense variants identified in this study (A) with those in patients with heterozygous deletions at 19q12-q13.11 encompassing the *TSHZ3* locus (B), and in *Tshz3*-null mutant mice (C) reported in the literature. A phenotypical overlap with respect to features marked in red, including developmental delay/intellectual disability, autism, epilepsy, multicystic dysplastic kidney, hydronephrosis, hydroureter, vesicoureteral reflux, genital tract anomalies, was notably. [1]Kulharya et al. 1998; [2]Malan et al. 2009; [3]Adalat et al. 2010; [4]Chowdhury et al. 2014; [5]Caubit et al. 2016; [6]Caubit et al. 2008; [7]Caubit et al. 2010; [8]Sanchez-Martin et al. 2021

REFERENCES

Adalat S, Bockenhauer D, Ledermann SE et al. (2010) Renal malformations associated with mutations of developmental genes: messages from the clinic. Pediatr Nephrol 25:2247–2255. https://doi.org/10.1007/s00467-010-1578-y

Barrie ES, Overwater E, van Haelst MM et al. (2020) Expanding the spectrum of CEP55-associated disease to viable phenotypes. Am J Med Genet A 182:1201–1208. https://doi.org/10.1002/ajmg.a.61512

Blackburn ATM, Bekheirnia N, Uma VC et al. (2019) DYRK1A-related intellectual disability: a syndrome associated with congenital anomalies of the kidney and urinary tract. Genet Med 21:2755–2764. https://doi.org/10.1038/s41436-019-0576-0

Bondeson ML, Ericson K, Gudmundsson S et al. (2017) A nonsense mutation in CEP55 defines a new locus for a Meckel-like syndrome, an autosomal recessive lethal fetal ciliopathy. Clin Genet 92:510–516. https://doi.org/10.1111/cge.13012

Caubit X, Lye CM, Martin E et al. (2008) Teashirt 3 is necessary for ureteral smooth muscle differentiation downstream of SHH and BMP4. Development 135:3301–3310. https://doi.org/10.1242/dev.022442

Caubit X, Thoby-Brisson M, Voituron N et al. (2010) Teashirt 3 regulates development of neurons involved in both respiratory rhythm and airflow control. J Neurosci 30:9465–9476. https://doi.org/10.1523/JNEUROSCI.1765-10.2010

Caubit X, Gubellini P, Andrieux J et al. (2016) TSHZ3 deletion causes an autism syndrome and defects in cortical projection neurons. Nat Genet 48:1359–1369. https://doi.org/10.1038/ng.3681

Connaughton DM, Kennedy C, Shril S et al. (2019) Monogenic causes of chronic kidney disease in adults. Kidney Int 95: 914–928. https://doi.org/10.1016/j.kint.2018.10.031

Connaughton DM, Dai R, Owen DJ et al. (2020) Mutations of the Transcriptional Corepressor ZMYM2 Cause Syndromic Urinary Tract Malformations. Am J Hum Genet 107:727–742. https://doi.org/10.1016/j.ajhg.2020.08.013

Dong S, Wang C, Li X et al. (2019) Noncoding rare variants of TBX6 in congenital anomalies of the kidney and urinary tract. Mol Genet Genomics 294:493–500. https://doi.org/10.1007/s00438-018-1522-6

Fabretti F, Tschernoster N, Erger F, et al. (2021) Expanding the Spectrum of FAT1 Nephropathies by Novel Mutations That Affect Hippo Signaling. Kidney Int Rep 6:1368–1378. https://doi.org/10.1016/j.ekir.2021.01.023

Fernandez-Prado R, Kanbay M, Ortiz A et al. (2019) Expanding congenital abnormalities of the kidney and urinary tract (CAKUT) genetics: basonuclin 2 (BNC2) and lower urinary tract obstruction. Ann Transl Med 7:S226. https://doi.org/10.21037/atm.2019.08.73

Heidet L, Morinière V, Henry C et al. (2017) Targeted Exome Sequencing Identifies *PBX1* as Involved in Monogenic Congenital Anomalies of the Kidney and Urinary Tract. J Am Soc Nephrol 28:2901–2914. https://doi.org/10.1681/ASN.2017010043

Jain S and Chen F (2019) Developmental pathology of congenital kidney and urinary tract anomalies. Clin Kidney J 12:382–399. https://doi.org/10.1093/ckj/sfy112

Kitzler TM, Schneider R, Kohl S et al. (2019) COL4A1 mutations as a potential novel cause of autosomal dominant CAKUT in humans. Hum Genet 138:1105–1115. https://doi.org/10.1007/s00439-019-02042-4

Kolvenbach CM, Dworschak GC, Frese S et al. (2019) Rare Variants in BNC2 Are Implicated in Autosomal-Dominant Congenital Lower Urinary-Tract Obstruction. Am J Hum Genet 104:994–1006. https://doi.org/10.1016/j.ajhg.2019.03.023

Kosfeld A, Martens H, Hennies I et al. (2018) Kongenitale Anomalien der Nieren und ableitenden Harnwege (CAKUT). medgen 30:448–460. https://doi.org/10.1007/s11825-018-0226-y

Kulharya AS, Michaelis RC, Norris KS et al. (1998) Constitutional del(19)(q12q13.1) in a three-year-old girl with severe phenotypic abnormalities affecting multiple organ systems. Am J Med Genet 77:391–394. https://doi.org/10.1002/(SICI)1096-8628(19980605)77:5<391::AID-AJMG7>3.0.CO;2-Q

Malan V, Raoul O, Firth HV et al. (2009) 19q13.11 deletion syndrome: a novel clinically recognisable genetic condition identified by array comparative genomic hybridisation. J Med Genet 46:635–640. https://doi.org/10.1136/jmg.2008.062034

Mann N, Kause F, Henze EK et al. (2019) CAKUT and Autonomic Dysfunction Caused by Acetylcholine Receptor Mutations. Am J Hum Genet 105:1286–1293. https://doi.org/10.1016/j.ajhg.2019.10.004

Moorman AF, Houweling AC, de Boer PA et al. (2001) Sensitive nonradioactive detection of mRNA in tissue sections: Novel application of the whole-mount in situ hybridization protocol. J Histochem Cytochem 49:1–8. https://doi.org/10.1177/002215540104900101

Nicolaou N, Pulit SL, Nijman IJ et al. (2016) Prioritization and burden analysis of rare variants in 208 candidate genes suggest they do not play a major role in CAKUT. Kidney Int 89:476–486. https://doi.org/10.1038/ki.2015.319

Rawlins LE, Jones H, Wenger O et al. (2019) An Amish founder variant consolidates disruption of CEP55 as a cause of hydranencephaly and renal dysplasia. Eur J Hum Genet 27:657–662. https://doi.org/10.1038/s41431-018-0306-0

Sanchez-Martin I, Magalhães P, Ranjzad P et al. (2021) Haploinsufficiency of the mouse Tshz3 gene leads to kidney defects. Hum Mol Genet 31:1921–1945. https://doi.org/10.1093/hmg/ddab362

Schneider CA, Rasband WS, Eliceiri KW (2012) NIH Image to ImageJ: 25 years of image analysis. Nat Methods 9:671–675. https://doi.org/10.1038/nmeth.2089

Szot JO, Campagnolo C, Cao Y et al. (2020) Bi-allelic Mutations in NADSYN1 Cause Multiple Organ Defects and Expand the Genotypic Spectrum of Congenital NAD Deficiency Disorders. Am J Hum Genet 106:129–136. https://doi.org/10.1016/j.ajhg.2019.12.006

van der Ven AT, Connaughton DM, Ityel H, et al. (2018a) Whole- Exome Sequencing Identifies Causative Mutations in Families with Congenital Anomalies of the Kidney and Urinary Tract. J Am Soc Nephrol 29:2348–2361. https://doi.org/10.1681/ASN.2017121265

van der Ven AT, Vivante A, Hildebrandt F (2018b) Novel Insights into the Pathogenesis of Monogenic Congenital Anomalies of the Kidney and Urinary Tract. J Am Soc Nephrol 29:36–50. https://doi.org/10.1681/ASN.2017050561

Vivante A and Hildebrandt F (2016) Exploring the genetic basis of early-onset chronic kidney disease. Nat Rev Nephrol 12:133–146. https://doi.org/10.1038/nrneph.2015.205

Yang N, Wu N, Dong S et al. (2020) Human and mouse studies establish TBX6 in Mendelian CAKUT and as a potential driver of kidney defects associated with the 16p11.2 microdeletion syndrome. Kidney Int 98:1020–1030. https://doi.org/10.1016/j.kint.2020.04.045
